# Rapidly Progressing Fungal Keratitis with Endophthalmitis Post SARS-CoV-2 Infection

**DOI:** 10.4269/ajtmh.21-1054

**Published:** 2022-03-04

**Authors:** Somasheila I. Murthy, Brijesh Takkar, Dilip Kumar Mishra

**Affiliations:** ^1^ Cornea Service, The Cornea Institute, L V Prasad Eye Institute, Kallam Anji Reddy Campus, Hyderabad, Telangana, India;; ^2^ Smt Kannuri Santhamma Center for Vitreoretinal Diseases, L V Prasad Eye Institute, Kallam Anji Reddy Campus, Hyderabad, India;; ^3^ Indian Health Outcomes, Public Health and Economics Research, Hyderabad, India;; ^4^ Ophthalmic Pathology Service, L V Prasad Eye Institute, Kallam Anji Reddy Campus, Hyderabad, India

**Figure 1.**
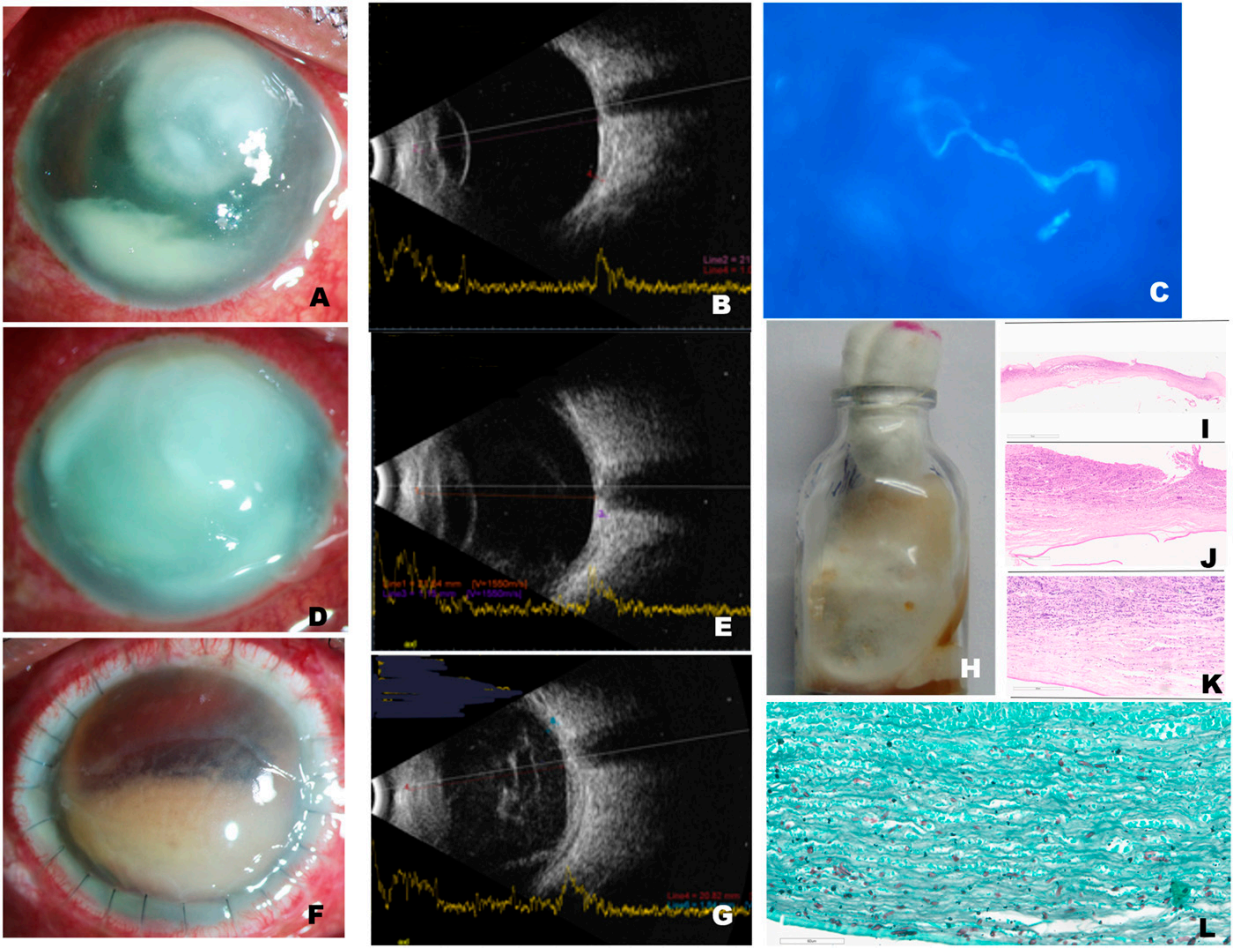
(**A**) Slit-lamp photograph (diffuse illumination) of the right eye at presentation shows a full-thickness, dry-looking infiltrated area measuring 6.5 × 5 mm. (**B**) Ultrasound shows clear vitreous cavity. (**C**) Microscopic evaluation of slides prepared from corneal scrapings shows septate fungal filaments under fluorescence microscope (potassium hydroxide with calcofluor white stain, ×400 magnification). (**D**) Three days later, the infection had progressed to involve the entire cornea. (**E**) Ultrasound at this visit shows very low-grade vitreous echoes. (**F**) Slit-lamp photograph 2 weeks after the first surgery shows graft edema, 24 sutures in situ, and blood-tinged pus in the anterior chamber (hypopyon). (**G**) Ultrasound at this visit shows only a very few vitreous echoes. (**H**) Half of the corneal specimen from the keratoplasty surgery was subjected to microbiology and inoculated on potato dextrose agar and incubated at 27°C for 2 weeks. It shows the growth of a velvety fungal colony, cream with an orange tinge, which was identified as *Fusarium solani*. (**I**) Histopathology of the other half of the corneal button shows ulceration with thinning on hematoxylin–eosin stain at low magnification (×2 magnification). (**J**) The same specimen under higher magnification and hematoxylin–eosin stain shows chronic inflammation (×10 magnification). (**K**) Thin septate fungal hyphae are noted on (**K**) periodic acid–Schiff and Gomori methenamine silver stain, (**L**) extending up to the Descemet’s membrane (×10 magnification). This figure appears in color at www.ajtmh.org.

**Figure 2.**
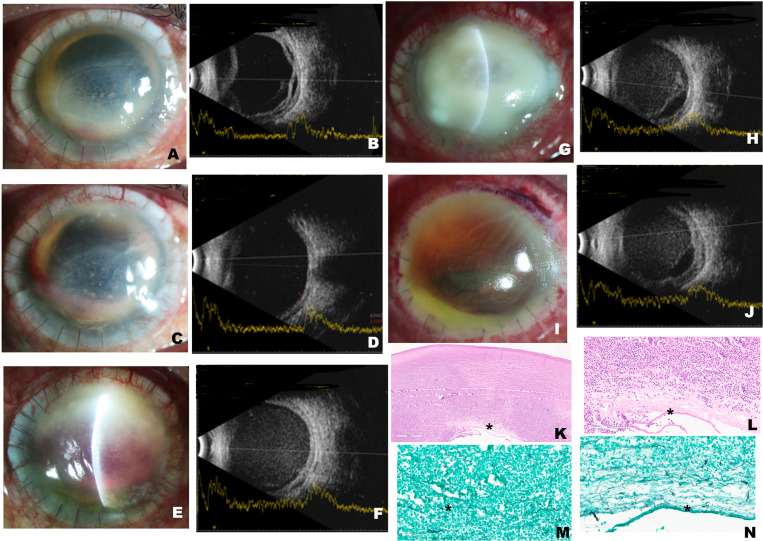
(**A**) Four weeks after the first surgery, there was improvement. Graft edema persisted, but the epithelial defect and hypopyon had both decreased. Topical steroids were started because the condition was better. (**B**) The corresponding ultrasound shows a clear vitreous cavity and a detached, thickened choroid. Five weeks later, there is (**C**) further retraction of the hypopyon and clearing of the graft, with (**D**) corresponding ultrasound showing decreased choroidal thickening. (**E**) Six weeks postoperatively, the slit-lamp photograph of the graft shows recurrence of the infection superiorly. (**F**) The corresponding ultrasound shows a uniform increase in vitreous echoes suggestive of vitreous hemorrhage. (**G**) A week later (7 weeks after surgery), the graft is completely infected. (**H**) The corresponding ultrasound shows exudates and vitreous hemorrhage. (**I**) Four weeks after the second transplant, there is dense edema of the graft and blood in the anterior chamber (hyphema), but no infection. (**J**) Ultrasound shows persistent vitreous hemorrhage. Histopathology of the second corneal specimen shows (**K**) edematous, densely infiltrated tissue with neutrophilic exudates (hematoxylin–eosin stain, ×10 magnification) and (**L**) Descemet fragmentation (asterisk) (hematoxylin–eosin stain, ×10 magnification). Gomori methenamine silver stain shows the presence of fungal filaments (asterisk) (**M**) at the posterior stroma in a background of necrosis (x10 magnification); these filaments are also noted at the level of the Descemet’s membrane (**N**) (asterisk) (x20 magnification). This figure appears in color at www.ajtmh.org.

A 56-year-old male farmer presented with a 1-week history of painful visual loss after trauma with a rice husk to his right eye. He had no history of diabetes or immune dysfunction. He was hospitalized a month previously for COVID-19 and had received five injections of intravenous methyl prednisolone (500 mg). At presentation, his visual acuity was perception of light. The cornea showed a full-thickness infection (Figure 1A). Ultrasonography showed a clear vitreous cavity (Figure 1B). Microbiology from corneal scrapings revealed fungal filaments (Figure 1C). Natamycin 5% eye drops hourly and oral ketoconazole 200 mg twice daily were started. However, the infection progressed rapidly (Figure 1D and E), and an urgent corneal transplant was done. Because the lens was also infected, cataract extraction was also performed (surgery 1). Despite this, the condition worsened over 2 weeks. The infection spread deeper to the posterior segment of the eye (endophthalmitis) (Figure 1F and G), necessitating vitrectomy and injections of amphotericin B (5 µg) and voriconazole (100 μg) (surgery 2). Corneal tissue grew *Fusarium solani* (Figure 1H). Histopathology showed deep invasion (Figure 1I–L). Over the next 2 weeks, there appeared to be improvement; hence, topical prednisolone acetate 1% was started (4 weeks after transplant or 2 weeks after vitrectomy) (Figure 2A–D). At 5 weeks post-transplant, the patient seemed better; but, suddenly at 6 weeks, he presented with recurrence of infection in the graft (Figure 2E–H). As a last effort, repeat transplantation with intraocular wash was performed (surgery 3). One month later (Figure 2 I and J), the infection had resolved but the eye was already shrinking (phthisis). Histopathology again revealed fungal filaments (Figure 2K–N).

Fungal keratitis can worsen rapidly when associated with predisposing factors such as trauma, topical corticosteroids use, or uncontrolled diabetes mellitus.^1^^,^^2^ Therapy for COVID-19 infection often includes systemic glucocorticoids, among other agents, for acute respiratory involvement.^3^ Our patient developed infection 1 month after having COVID-19. His condition worsened despite aggressive medical and surgical management. Decreased production of CD4+ T cells and CD8+ T cells, and decreased cytokines in COVID-19 have been associated with systemic immunosuppression, predisposing to secondary opportunistic infections (especially fungal).^4^^,^^5^ *Fusarium* infection itself is a poor prognostic factor because it is known to progress to endophthalmitis, which invariably has a poor outcome.^2^ We postulate that the weakened host immunity and the use of glucocorticoids in COVID-19 may have been an additional risk factor for the poor outcome in our patient.
